# Association between serum uric acid levels and peripheral artery disease in Chinese adults with hypertension

**DOI:** 10.3389/fendo.2023.1197628

**Published:** 2023-08-22

**Authors:** Fengyu Han, Chao Yu, Feng Hu, Wei Zhou, Tao Wang, Linjuan Zhu, Xiao Huang, Huihui Bao, Xiaoshu Cheng

**Affiliations:** ^1^ Department of Cardiovascular Medicine, The Second Affiliated Hospital of Nanchang University, Nanchang, Jiangxi, China; ^2^ Jiangxi Provincial Cardiovascular Disease Clinical Medical Research Center, Nanchang, Jiangxi, China; ^3^ Jiangxi Sub-center of National Clinical Research Center for Cardiovascular Diseases, Nanchang, Jiangxi, China; ^4^ Center for Prevention and Treatment of Cardiovascular Diseases, The Second Affiliated Hospital of Nanchang University, Nanchang, Jiangxi, China

**Keywords:** serum uric acid, peripheral artery disease, hyperuricemia, hypertension, adult

## Abstract

**Background:**

Higher serum uric acid (SUA) can cause gout, which is principally characterized by arthritis due to monosodium urate crystal deposition in the lower extremities. High levels of SUA have been linked to endothelial dysfunction, oxidative stress, and inflammation, all of which are involved in the pathogenesis of peripheral artery disease(PAD). To date, the relationship between SUA levels and PAD is still poorly understood.

**Method:**

An analysis of 9,839 Chinese adults with essential hypertension from the ongoing China H-type Hypertension Registry Study was conducted in this cross-sectional study. Patients with an ABI ≤0.9 was diagnosed with PAD. Hyperuricemia was defined as SUA levels >420 mol/L in men and >360 mol/L in women. The association between SUA levels and PAD was evaluated using multivariable logistic regression models based on odds ratios (ORs) and their 95% confidence intervals (CIs).

**Results:**

The enrolled subjects ranged in age from 27 to 93 years, with a mean age of 63.14 ± 8.99 years. The proportion of male patients was 46.22%, and the prevalence of hyperuricemia was 50.72%. In males, hyperuricemia was positively associated with the risk of PAD (adjusted OR per SD increase: 1.72, 95% CI 1.17 to 2.53, P =0.006). Males in the highest SUA tertile were significantly more likely to have PAD (adjusted OR: 2.63, 95% CI 1.42 to 4.86, P = 0.002; P for trend = 0.001). However, this positive relationship was not observed in females (adjusted OR: 1.29, 95% CI 0.77 to 2.17, P = 0.327; P for trend = 0.347).

**Conclusion:**

According to this cross-sectional study, higher SUA levels were positively associated with PAD in male hypertensive patients, while this positive relationship disappeared in female participants.

## Introduction

Peripheral artery disease (PAD) is a circulatory condition that affects the blood vessels outside of the heart and brain, most commonly in the legs. It occurs when the arteries that supply oxygen and nutrients to the muscles of the legs become narrowed or blocked by an accumulation of fatty deposits or plaque, a process called atherosclerosis. The reduced blood flow can cause symptoms such as claudication, ischemic rest pain, numbness, and cramping, which can interfere with mobility and reduce the quality of life. Besides, PAD can also cause sores or ulcers on the legs or even lead to tissue loss and amputation (1). The presence of PAD is usually diagnosed via ankle-brachial index (ABI), which is calculated by dividing the ankle systolic pressure by the brachial systolic pressure with a sphygmomanometer or ultrasound Doppler device. Previous studies showed that several cardiovascular events might be linked to PAD, including atrial fibrillation, acute myocardial infarction, stroke, and death (2–4). However, the perniciousness of PAD was underestimated on account of its asymptomatic manifestation in the early stage, and the biomarkers that may identify patients with new-onset PAD or individuals at higher risk of PAD are urgently needed (5).

The purine nucleotide cycle produces serum uric acid (SUA). Hyperuricemia (HUA), or elevated serum uric acid, is a metabolic disorder caused by purine metabolism disorders, excessive uric acid production, or reduced excretion (6).On this basis, HUA could further cause gout, which is principally characterized by arthritis due to monosodium urate crystal deposition in the lower extremities (7).

HUA has also been associated with subclinical atherosclerosis, markers of inflammation, oxidative stress, and endothelial dysfunction according to the current literature, all of which are involved in the pathogenesis of PAD (8). Numerous investigations have demonstrated that HUA is an independent risk factor for the occurrence and prevalence of several diseases, including diabetes, heart disease, hypertension, and chronic kidney disease (9–12). Therefore, HUA may contribute to the development or progression of PAD by promoting atherosclerosis, inflammation, and endothelial dysfunction, which are all key pathological mechanisms involved in the development of PAD. However, further research is needed to fully elucidate the possible association between HUA or elevated SUA and PAD.

To date, research on the relationship between PAD and SUA or community-based studies has been limited (13). Researchers found that HUA is an independent risk factor for carotid plaque in men without metabolic syndrome in a cross-sectional study (14). Yoko Sotoda et al. demonstrated that HUA was associated with leg ischemia in patients with PAD, independent of other atherosclerotic risk factors (15). However, most of this research aimed to investigate the relationship between PAD and SUA in a common population. To explore the relationship between SUA and PAD in hypertensive patients, we conducted this real-world, multi-center, observational study in South China in March 2018.

## Methods

### Study design and participants

This study utilized the baseline data from the China H-type Hypertension Registry Study (Registration number: ChiCTR1800017274), which was a prospective, real-world, and observational study. Data collection methods and exclusion criteria have been described previously (16). A protocol for the study was approved by the Ethics Committee of the Institute of Biomedicine, Anhui Medical University (No. CH1059), in accordance with the Declaration of Helsinki. Forms of informed consent were completed by all participants before enrollment.

**Figure 1:**
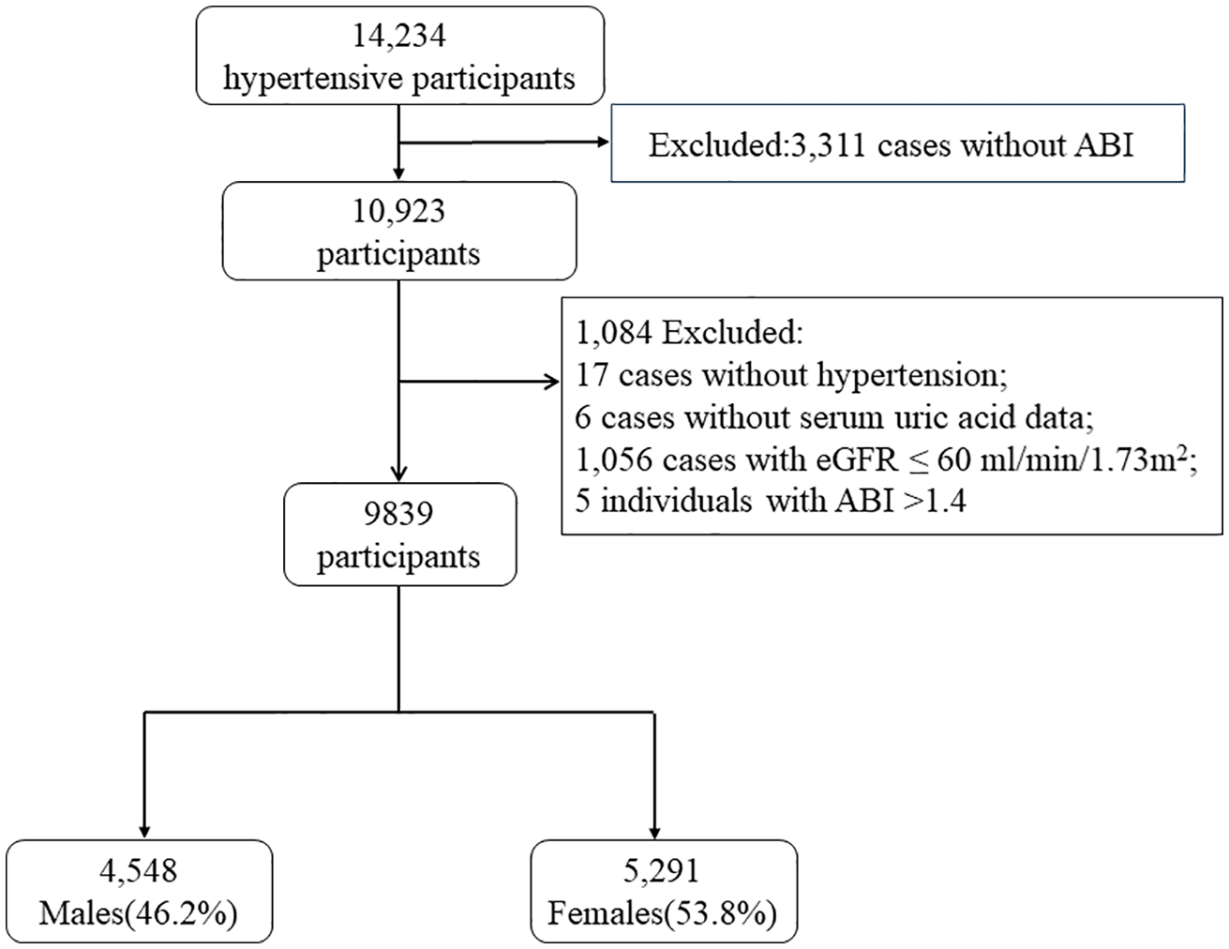
Flow chart of the study population. ABI, ankle brachial index; PAD, peripheral arterial disease; SUA, serum uric acid; eGFR, estimated glomerular filtration rate.

A total of 10, 923 participants completed the ankle brachial index (ABI) measurement in this study. After excluding 17 individuals without hypertension, 6 cases without serum uric acid data, 1,056 cases with eGFR ≤ 60 ml/min/1.73m^2^, and 5 individuals with ABI >1.4 (17, 18), finally 9, 839 participants were enrolled in our analysis (**Figure 1**).

### Data collection and indexes determination

In this study, researchers utilized carefully compiled questionnaires to collect data on a variety of participant characteristics, including demographic information such as age and gender, lifestyle habits like smoking and drinking, medical history (atrial fibrillation, hypertension, diabetes mellitus, and dyslipidemia), as well as medication use including antihypertensive, hypoglycemic, lipid-lowering, and antiplatelet agents. Current smokers were defined as participants who smoked at least 20 packets of cigarettes in their lifetime and currently smoke cigarettes. Former smokers were participants who had smoked at least 20 packets of cigarettes in their lifetime, and quit smoking for at least 1 month. A drinker was defined as someone who has consumed at least one alcoholic beverage per week in the past month (19).

Additionally, we collected participants’ anthropometric measurements, including weight, height, waist circumference, hip circumference, systolic blood pressure (SBP), diastolic blood (DBP), and heart rate (HR), as well as their height and waist circumference measurements. A validated upper arm medical electronic sphygmomanometer (HBP-1300; Omron; Kyoto, Japan) was utilized with the appropriate cuff size for the upper right arm to measure blood pressure. For each participant, four consecutive office blood pressure measurements were performed, and the mean of the last three readings was analyzed. A 1.5-m-long inelastic measuring tape with 0.1 cm resolution was used to measure waist circumference and hip circumference. Body weight without heavy clothing was measured using a body fat and weight measurement device (V-body HBF-371, Omron; Kyoto, Japan). BMI was calculated by multiplying weight (in kilograms) by height (in meters squared: kg/m2). The waist-hip ratio (WHR) was calculated by dividing the waist circumference by the hip circumference. The mean values of the last three readings were analyzed.

Participants were classified into three groups based on their BMI values: underweight (<24 kg/m2), overweight (24-28 kg/m2), and obese (≥28 kg/m2), using published standards for the Chinese population. Central obesity was defined as male participants with a WHR of ≥0.9 or female participants with a WHR of ≥0.85 (20).

After an overnight fast of at least 12 hours, blood samples were collected by venipuncture. Blood biochemical tests for plasma total homocysteine (tHcy), fasting blood glucose (FBG), total cholesterol (TC), total triglyceride (TG), high-density lipoprotein cholesterol (HDL-C), low-density lipoprotein cholesterol (LDL-C), SUA, serum creatinine, blood urea nitrogen (BUN), total and direct bilirubin, aspartate aminotransferase (AST), and alanine aminotransferase (ALT) were measured using automatic clinical analyzers (Beckman Coulter) at the core laboratory of the National Clinical Research Center for Kidney Disease, Guangzhou, China. In our study, DM was defined as FBG levels above 7.0 mmol/L or treatment for diagnosed DM (21). Dyslipidemia was defined as having TG >2.3 mmol/L, TC >6.2 mmol/L, LDL >4.1 mmol/L, HDL <1.0 mmol/L in men and <1.2mmol/L in women (22), or being treated with appropriate lipid-lowering agents. HUA was defined as SUA levels >420 mol/L in men and >360 mol/L in women by most studies among Chinese populations (23, 24). In some studies, HUA was designated with lower cutoff points (25, 26).CKD-EPI’s (Chronic Kidney Disease Epidemiology Collaboration) equation was used to estimate glomerular filtration rate (eGFR) (27).

### Measurement of ABI and definition of PAD

The ABI was performed using a BP-203 RPE III networked arteriosclerosis detection device (VP-2000, Omron Health Care, Kyoto, Japan) following the guidelines recommended by the American College of Cardiology/American Heart Association (28). Participants rested in a quiet room for at least five minutes in a supine position before undergoing ABI measurements. Consumption of coffee, tea, cigarettes, or alcohol was prohibited for 30 minutes prior to the test. Electrodes were attached to the participant’s forearms to measure an electrocardiogram (ECG). Both arms (brachial artery) and ankles (posterior tibial artery) were fitted with a blood pressure cuff and a Doppler ultrasound device. The ABI was measured for a period of 10-30 seconds, and the lowest ABI value between right and left was used as a normality parameter. Patients with an ABI ≤0.9 was diagnosed as PAD (17, 18, 28–30). Patients with diabetes and/or advanced chronic kidney disease are more likely to have ABI values >1.40, indicating that the arteries cannot be compressed. Only five patients with ABI values higher than 1.4 were excluded at the entrance.

### Statistical analysis

The clinical characteristics of participants were determined based on their baseline SUA levels. A continuous variable was expressed as its mean ± standard deviation (SD) or median (Q1-Q3). In both groups, variables with normal distributions and homoscedasticity were analyzed using a one-way analysis of variance. The Kruskal-Wallis test was performed on variables that showed skewed distribution. A chi-square or Fisher’s exact probability test was used to compare groups based on categorical variables expressed as counts (percentages).

The association between SUA levels and PAD was evaluated using multivariable logistic regression models based on odds ratios (ORs) and their 95% confidence intervals (CIs). Covariates were not taken into account in the crude model. Age was the only adjustment made to Model I. Model II was a covariate model. The covariate model screened for covariates including age, SBP, DBP, BMI, smoking and drinking status, homocysteine, TC, TG, HDL-C, LDL-C, AST, ALT, serum creatinine, DM, antihypertensive agents, lipid-lowering agents, and antiplatelet agents. **Supplementary Table 1** showed the associations of each covariate with PAD. Covariates were the main model. Subjects were categorized into SUA tertiles, and SUA predictive capacity was explored for SUA, with either considered as a continuous or a categorical variable.

Finally, to ensure the robustness between SUA levels and PAD in different genders, the subgroup analysis was presented in tabular form with a forest plot using stratified multivariate regression and interaction analyses.

The statistical package R (The R Foundation; http://www.r-project.org; version 4.2.0) was used to perform all statistical analyses and EmpowerStats (R; www.empowerstats.com; X&Y Solutions, Inc, Boston, MA, USA; version 4.2). Statistical significance was denoted by a P value of <0.05, and each P value is two-tailed.

## Results

### Characteristics of the population studied

This study enrolled 9,839 Chinese adults with hypertension, with ages ranging from 27 to 93 years and a mean age of 63.14 ± 8.99. **Table 1** presents the clinical characteristics of the study participants, grouped by HUAAs the schematics revealed, the proportion of male participants was 46.22%, and the prevalence of HUA was 50.72%.

**Table 1:** Clinical characteristics of the study population grouped by HUA.

Characteristics	Total subjects	HUA		*P-value*
		No	Yes	
Number of subjects (n)	9839	4849 (49.28%)	4990 (50.72%)	
Gender (n)				<0.001
Male	4548	1964 (40.50%)	2584 (51.78%)	
Female	5291	2885 (59.50%)	2406 (48.22%)	
Age (years)	63.14 ± 8.99	63.19 ± 8.85	63.09 ± 9.12	0.58
SBP (mmHg)	148.46 ± 17.56	149.31 ± 17.43	147.63 ± 17.64	<0.001
DBP (mmHg)	89.38 ± 10.55	89.06 ± 10.38	89.69 ± 10.72	0.003
HR (times/min)	76.31 ± 13.99	76.46 ± 14.28	76.16 ± 13.70	0.282
BMI (kg/m^^2^)	23.16 ± 4.02	23.16 ± 4.02	24.14 ± 3.60	<0.001
BMI group (kg/m^^2^)				<0.001
Control (<24)	5517 (56.07%)	3041 (62.71%)	2476 (49.62%)	
Overweight (≥24, <28)	3304 (33.58%)	1447 (29.84%)	1857 (37.21%)	
General obesity (≥28)	1018 (10.35%)	361 (7.44%)	657 (13.17%)	
WHR	0.91 ± 0.18	0.90 ± 0.25	0.92 ± 0.07	<0.001
Smoking status, n(%)				<0.001
Never	5743 (58.38%)	3019 (62.26%)	2724 (54.60%)	
Former	1510 (15.35%)	643 (13.26%)	867 (17.38%)	
Current	2585 (26.28%)	1187 (24.48%)	1398 (28.02%)	
Drinking status, n(%)				<0.001
Never	6135 (62.37%)	3203 (66.07%)	2932 (58.77%)	
Former	1397 (14.20%)	730 (15.06%)	667 (13.37%)	
Current	2305 (23.43%)	915 (18.87%)	1390 (27.86%)	
Hcy (μmol/L)	17.19 ± 10.17	16.20 ± 9.45	18.15 ± 10.74	<0.001
TC (mmol/L)	5.16 ± 1.10	5.06 ± 1.05	5.26 ± 1.13	<0.001
TG (mmol/L)	1.78 ± 1.25	1.57 ± 1.01	1.99 ± 1.42	<0.001
HDL-C (mmol/L)	1.60 ± 0.43	1.62 ± 0.44	1.57 ± 0.43	<0.001
LDL-C (mmol/L)	3.00 ± 0.80	2.92 ± 0.78	3.08 ± 0.82	<0.001
SUA (umol/L)	402.79 ± 113.72	316.48 ± 57.36	486.67 ± 89.59	<0.001
HUA, n(%)	4990 (50.72%)	2584 (56.82%)	2406 (45.47%)	<0.001
BUN (mmol/L)	5.25 ± 1.46	5.13 ± 1.41	5.36 ± 1.50	<0.001
Serum creatinine (mmol/L)	64.12 ± 17.23	58.30 ± 15.70	69.78 ± 16.76	<0.001
eGFR (ml/min/1.73m^2^)	93.37 ± 14.59	97.62 ± 13.57	89.25 ± 14.37	<0.001
Total bilirubin (mmol/L)	14.82 ± 6.94	14.48 ± 7.18	15.14 ± 6.69	<0.001
Direct bilirubin (mmol/L)	5.66 ± 2.71	5.64 ± 3.16	5.69 ± 2.18	0.388
AST (U/L)	26.80 ± 16.91	25.26 ± 19.22	28.30 ± 14.14	<0.001
ALT (U/L)	20.62 ± 17.10	18.61 ± 18.56	22.58 ± 15.29	<0.001
DM, n(%)	1723 (17.51%)	764 (15.76%)	959 (19.22%)	<0.001
Dyslipidemia, n(%)	3598 (36.57%)	1456 (30.03%)	2142 (42.93%)	<0.001
Atrial fibrillation, n(%)	252 (2.56%)	100 (2.06%)	152 (3.05%)	0.002
Antihypertensive agents, n(%)	6338 (64.42%)	3046 (62.82%)	3292 (65.99%)	0.001
Hypoglycemic agents, n(%)	482 (4.90%)	245 (5.05%)	237 (4.75%)	0.486
Lipid-lowering agents, n(%)	324 (3.29%)	167 (3.44%)	157 (3.15%)	0.408
Antiplatelet agents, n(%)	368 (3.74%)	181 (3.73%)	187 (3.75%)	0.969
Ankle brachial index	1.09 ± 0.09	1.09 ± 0.09	1.10 ± 0.10	0.032
PAD, n(%)	263 (2.67%)	109 (2.25%)	154 (3.09%)	0.01

SBP, systolic blood pressure; DBP, diastolic blood pressure; HR, heart rate; BMI, body mass index; WHR, waist hip rate; Hcy, homocysteine; FBG, fasting blood glucose; TC, total cholesterol; TG, total triglyceride; HDL-C, high-density lipoprotein cholesterol; LDL-C, low-density lipoprotein cholesterol; SUA, serum uric acid; BUN, blood urea nitrogen; eGFR, estimated glomerular filtration rate; AST, aspartate aminotransferase; ALT, alanine aminotransferase; DM, diabetes mellitus; PAD, peripheral arterial disease.

Study participants were grouped by sex and their clinical characteristics are summarized in **Supplementary Table 2**. The overall prevalence of PAD was 2.67%, with male participants exhibiting a higher proportion (3.17%) than female participants (2.25%). The proportion of HUA was higher in male (56.82%) than in female participants (45.47%). Furthermore, as shown in **Supplementary Table 2**, we found significant differences between male and female participants with regard to current smoking (50.53% vs. 5.43%) and drinking habits (43.68% vs. 6.03%). No significant difference was observed in medication use, including antihypertensive agents and lipid-lowering agents, between the two genders. **Supplementary Table 2** also illustrates that women had a higher use rate of hypoglycemic agents (5.44%) compared to men (4.27%), while men had a higher use rate of antiplatelet agents (4.20%) compared to women (3.35%), with statistical significance (P = 0.007 and P = 0.026, respectively). Specifically, male patients exhibited a higher likelihood of having elevated levels of DBP, WHR, Hcy, SUA, BUN, serum creatinine, eGFR, total and direct bilirubin, AST, ALT, and ABI. Conversely, female patients exhibited lower levels of SBP, BMI, FBG, TC, TG, HDL-C, and LDL-C.

### Association between SUA levels and PAD in the hypertensive population

To assess the association between SUA levels and PAD, multivariable logistic regression was conducted after adjusting for covariates that could impact the outcome by more than 10%. Results showed no significant association between SUA levels and PAD risk when SUA was analyzed as a continuous variable in the whole sample (adjusted OR per SD increase: 1.00, 95% CI 1.00 to 1.00, P = 0.055; **Supplementary Table 3**).

Multivariable logistic regression analyses were then performed separately for male and female particiants. There was no significant association between SUA levels and PAD risk when SUA levels were viewed as continuous variables in both men (adjusted OR per SD increase: 1.19, 95% CI 0.98 to 1.45, P = 0.087; **Table 2**) and women (adjusted OR per SD increase: 1.15, 95% CI 0.91 to 1.46, P = 0.232; **Table 2**). Furthermore, multivariable logistic regression analyses were conducted to examine the relationship between PAD and hyperuricemia, which was defined as SUA > 420 μmol/L in men and > 360 μmol/L in women. A positive association was found between hyperuricemia and PAD risk in men (adjusted OR per SD increase: 1.72, 95% CI 1.17 to 2.53, P = 0.006; **Table 2**). However, when HUA was defined as SUA > 5.1 mg/dL for female and 5.6 mg/dL for male participants, we did not observe a significant relationship between HUA and PAD in the overall population (adjusted OR per SD increase:1.30, 95% CI: 0.91 to 1.87, p = 0.147, **Supplementary Table 4**). This lack of association was consistent when analyzing male participants separately (adjusted OR per SD increase: 1.78, 95% CI: 0.97 to 3.25, p = 0.062; **Supplementary Table 5**) and female participants separately (adjusted OR per SD increase: 1.00, 95% CI: 0.63 to 1.60, p = 0.991; **Supplementary Table 5**). The highest SUA tertile was associated with a significantly higher prevalence of PAD compared to the lowest tertile in men (adjusted OR: 2.63, 95% CI 1.42 to 4.86, P = 0.002; P for trend = 0.001). However, this relationship was not significant in women (adjusted OR: 1.29, 95% CI: 0.77 to 2.17, P = 0.327; P for trend = 0.347).

**Table 2:** Hazard ratios of serum uric acid level categories for PAD by sex in different models.

Variables	Event, n(%)	Crude Model		Model I		Model II	
		HR (95%CI)	P-value	HR (95%CI)	P-value	HR (95%CI)	P-value
Male							
SUA							
Per SD μmol/L increase	144 (3.17%)	1.06 (0.90, 1.25)	0.502	1.13 (0.95, 1.34)	0.171	1.19 (0.98, 1.45)	0.087^a^
HUA							
No	51 (2.60%)	*Ref*		*Ref*		*Ref*	
Yes	93 (3.60%)	1.40 (0.99, 1.98)	0.057	1.54 (1.09, 2.19)	0.016	1.72 (1.17, 2.53)	0.006^a^
Tertiles of SUA							
T1 [38.00, 344.00]	15 (2.07%)	*Ref*		Ref		Ref	
T2 [345.00, 439.00]	45 (2.87%)	1.40 (0.77, 2.52)	0.267	1.42 (0.78, 2.58)	0.245	1.65 (0.89, 3.05)	0.114^a^
T3 [440.00, 1056.00]	84 (3.73%)	1.83 (1.05, 3.19)	0.033	2.05 (1.17, 3.59)	0.012	2.63 (1.42, 4.86)	0.002^a^
P for trend		0.019		0.004		0.001	
Female							
SUA							
Per SD μmol/L increase	119 (2.25%)	1.26 (1.03, 1.53)	0.022	1.23 (1.01, 1.51)	0.041	1.15 (0.91, 1.46)	0.232^b^
HUA							
No	58 (2.01%)	*Ref*		*Ref*		*Ref*	
Yes	61 (2.54%)	1.27 (0.88, 1.82)	0.201	1.20 (0.83, 1.73)	0.323	1.03 (0.69, 1.55)	0.881^c^
Tertiles of SUA							
T1 [108.00, 344.00]	49 (1.93%)	*Ref*		*Ref*		*Ref*	
T2 [345.00, 439.00]	39 (2.26%)	1.17 (0.77, 1.79)	0.461	1.10 (0.72, 1.69)	0.653	0.97 (0.62, 1.52)	0.892^d^
T3 [440.00, 879.00]	31 (3.00%)	1.57 (0.99, 2.47)	0.054	1.49 (0.94, 2.35)	0.088	1.29 (0.77, 2.17)	0.327^d^
P for trend		0.056		0.096		0.347	

PAD, peripheral arterial disease; SUA, serum uric acid; HUA, hyperuricemia; Ref, reference; HR, hazard ratio; CI, confidence interval; SD, standard deviation.

Model I adjusted for age.

Model II: a.adjusted for age, SBP, DBP, BMI, smoking and drinking status, Hcy, TG, LDL-C, serum creatinine, ALT, AST, antihypertensive agents, and antiplatelet agents.

b.adjusted for age, SBP, DBP, BMI, Hcy, TG, LDL-C, serum creatinine, and antiplatelet agents.

c.adjusted for age, SBP, DBP, BMI, Hcy, TG, HDL-C, LDL-C, serum creatinine, ALT, AST, antihypertensive agents, lipid-lowering agents, and antiplatelet agents.

d.adjusted for age, SBP, DBP, BMI, Hcy, TG, HDL-C, LDL-C, serum creatinine, ALT, AST, and antihypertensive agents.

### Subgroup analyses by potential effect modifiers

To explore whether the association between SUA levels and the prevalence of PAD was still stable in different subgroups, subgroup analysis was presented in tabular form with a forest plot using stratified multivariate regression and interaction analyses.

In any subgroup of male patients, there were no statistically significant interactions, including age (<60 vs. ≥60 years), SBP dichotomy (≤146.67 vs. ≥ 147.00 mmHg), DBP dichotomy (≤89.33 vs. ≥89.67 mmHg), different BMI group, smoking habit (no vs. yes), drinking habit (no vs. yes), antihypertensive agents (no vs. yes), homocysteine (<15 vs. ≥15 μmol/L), LDL dichotomy (< 2.93 vs. ≥2.94 mmol/L), and serum creatinine (≤62.0 vs. ≥63.0 mmol/L) (all P for interactions >0.05; **Supplementary Figure 1**).

Based on the subgroup analysis, none of the subgroups had statistically significant interactions for females, including age (<60 vs. ≥60 years), the SBP dichotomy (≤146.67 vs. ≥ 147.00 mmHg), the DBP dichotomy (≤89.33 vs. ≥89.67 mmHg), different BMI group, antihypertensive agents (no vs. yes), antiplatelet agents (no vs. yes), homocysteine (<15 vs. ≥15 μmol/L), LDL dichotomy (< 2.94 vs. ≥2.94 mmol/L), and AST dichotomy (≤23.00 vs. ≥24.00 U/L) (all P for interactions >0.05; **Supplementary Figure 2**).

## Discussion

This study investigated the association between PAD and SUA levels in hypertensive patients in South China. Higher levels of SUA might be positively associated with PAD after being adjusted for major cardiovascular risk factors in male patients with hypertension.

Numerous investigations have delved into the conceivable correlation between SUA levels and PAD. However, the outcomes have been inconsistent due to variations in the populations studied (31–34). The data regarding whether this connection is restricted to gender (male or female) or affects both remains inconclusive. Some research supported the results of our study. A cohort study scrutinized serum and 24-hour SUA levels and additional risk factors among two groups of hypertensive patients: 145 lacking PAD and 166 with PAD (35). This paper revealed that, in essential hypertensive patients, a higher level of SUA values was associated with worse peripheral circulatory function and was more pronounced in those with PAD. In 2008, Shankar Anoop and colleagues demonstrated a noteworthy correlation between SUA levels and PAD in both genders in a nationally representative sample of the US population (13). Furthermore, there was evidence suggesting that an association between SUA levels and atherosclerosis was plausible (36, 37). A cross-sectional study corroborated that higher SUA levels might be positively associated with leg ischemia in male patients with PAD (15). However, the small sample size of only 87 male participants at enrollment precludes definitive conclusions concerning any gender differences in the relationship between SUA and PAD. Dong Jing et al. discovered that elevated SUA was an independent risk factor for developing new onset hypertension in a cohort, single-center study (38). Combined with the conclusion of this analysis, it is hypothesized that the relationship between SUA and PAD is mediated by hypertension.

Several prior experimental studies have investigated the mechanisms by which elevated SUA levels are associated with hypertension, stroke, atrial fibrillation, and other cardiovascular diseases (7, 38–41). SUA is directly or circumstantially associated with inflammation, arterial stiffness, renal function decline, smooth muscle proliferation, and endothelial dysfunction. These inflammatory mediators also play a critical role in peripheral vasculature (42, 43). SUA has been reported to engulf smooth muscle proliferation through NLRP3 (Nod-like receptor family protein 3) (44, 45). Furthermore, SUA restrained the activity and phosphorylation of AMPK(AMP-activated protein kinase). A decrease in AMPK led to the activation of NLRP3 inflammasomes (36). Then the activation of NLRP3 further led to an increase of inflammatory markers such as interleukin-1 and interleukin-18. HUA activated the renin-angiotensin system, reduced endothelial nitric oxide bioavailability, simulated oxidative stress, and enhanced arterial stiffness (45, 46). A recent experiment declared that SUA might induce an increase in the Tissure Factor, which was the key initiator of the coagulation cascade (47). Nevertheless, the pathological mechanisms underlying the association between SUA levels and PAD remain incompletely elucidated to date. One potential explanation for this association is the influence of metabolic syndrome (48). Previous studies have discovered that higher SUA levels are strongly associated with metabolic syndrome, and HUA has been identified as a contributing risk factor within the context of multifactorial syndrome (49, 50). It is plausible that elevated uric acid levels represent a compensatory response to oxidative stress, as supported by previous research findings (51).

The main strength of our current study is that we collected a representative sample from southern China. This is the first study to elucidate the relationship between SUA and PAD in hypertensive patients in South China. To enhance the dependability of our study, subgroup analyses were conducted in addition to examining the overall relationship between SUA and PAD.

This study had some limitations that need to be further explained. First, given the cross-sectional nature of our study, we could not draw definitive conclusions about the causal relationship between SUA and PAD. Second, the limited scope of our study’s population, which was confined to South China, may restrict the generalizability of our findings. Last but not least, the history of some therapies, such as revascularization (52), the treatment with diuretics (31), and uric acid-lowering drugs, was not captured at enrollment, which might be covariates in the multivariable logistic regression. Given the present limitations, caution should be exercised in interpreting the findings of this cross-sectional study.

## Conclusion

In conclusion, higher SUA levels were positively associated with PAD in male hypertensive patients. We ascertained that elevated SUA levels were positively associated with an increased risk of PAD in the male hypertensives in this cross-sectional study. However, this positive correlation was not present in female patients with hypertension. Based on this cross-sectional study, further longitudinal, perspective, or multi-central studies are needed to determine the chronological or causal relationship between SUA and PAD in a hypertensive population.

## Data availability statement

The original contributions presented in the study are included in the article/**Supplementary material**. Further inquiries can be directed to the corresponding author.

## Ethics statement

The studies involving human participants were reviewed and approved by the Ethics Committee of the Institute of Biomedicine, Anhui Medical University. The patients/participants provided their written informed consent to participate in this study. Written informed consent was obtained from the individual(s) for the publication of any potentially identifiable images or data included in this article.

## Author contributions

FYH designed and executed the experiments, performed data analysis, and wrote the initial draft of the manuscript. CY designed and conducted the experiments, analyzed the data, and wrote the majority of the Methods section, and provided critical feedback and revisions throughout the writing process. XH, WZ, TW, and LZ conducted literature review, contributed to the manuscript’s organization, and provided editorial assistance. FH provided technical support for data analysis and critically reviewed the manuscript. HB and XC conceptualized the study, secured funding, and oversaw all aspects of the project. All authors contributed to the article and approved the submitted version.
