# Supplementary material for: Association between serum uric acid levels and peripheral artery disease in Chinese adults with hypertension
Source: Front Endocrinol (Lausanne). 2023 Aug 22;14:1197628. doi: 10.3389/fendo.2023.1197628 (PMC10478083; doi:10.3389/fendo.2023.1197628)
Supplement: Supplementary file 1 [file DataSheet_1.pdf]

Supplementary Table 1. Associations of covariates with peripheral arterial disease

Covariates	Male		Female	
	HR (95%CI)	*P*-value	*HR (95%CI)*	*P*-value
Age (years)	1.09 (1.07, 1.12)	<0.001	1.06 (1.04, 1.09)	<0.001
SBP (mmHg)	1.01 (1.00, 1.02)	0.023	1.02 (1.01, 1.03)	<0.001
DBP (mmHg)	0.96 (0.94, 0.97)	<0.001	0.97 (0.95, 0.99)	0.002
BMI (kg/m^^{2})	0.90 (0.85, 0.94)	<0.001	0.97 (0.93, 1.02)	0.304
Smoking status, n(%)				
Never	*Ref*			
Former	1.10 (0.63, 1.92)	0.733	1.51 (0.65, 3.48)	0.336
Current	1.81 (1.12, 2.93)	0.016	3.31 (1.97, 5.57)	<0.001
Drinking status, n(%)				
Never	*Ref*			
Former	0.89 (0.58, 1.37)	0.604	0.96 (0.50, 1.84)	0.893
Current	0.59 (0.40, 0.87)	0.007	0.97 (0.45, 2.10)	0.937
Hcy (μmol/L)	1.02 (1.01, 1.03)	<0.001	1.02 (1.00, 1.04)	0.067
TC (mmol/L)	1.07 (0.92, 1.26)	0.373	1.12 (0.95, 1.31)	0.171
TG (mmol/L)	0.88 (0.75, 1.04)	0.137	0.94 (0.80, 1.11)	0.465
HDL-C (mmol/L)	0.80 (0.54, 1.18)	0.264	0.75 (0.48, 1.17)	0.203
LDL-C (mmol/L)	1.20 (0.98, 1.48)	0.078	1.28 (1.04, 1.57)	0.022
Serum creatinine (mmol/L)	1.01 (1.00, 1.02)	0.051	1.02 (1.01, 1.04)	0.004
AST (U/L)	0.96 (0.94, 0.99)	0.001	0.99 (0.97, 1.01)	0.374
ALT (U/L)	0.96 (0.94, 0.98)	<0.001	0.99 (0.98, 1.01)	0.369
DM, n(%)				
No	*Ref*		*Ref*	
Yes	0.91 (0.57, 1.45)	0.682	1.14 (0.73, 1.78)	0.568
Antihypertensive agents, n(%)				
No	*Ref*		*Ref*	
Yes	2.36 (1.31, 4.25)	0.004	2.13 (1.02, 4.44)	0.043
Lipid-lowering agents, n(%)				
No	*Ref*		*Ref*	
Yes	1.25 (0.54, 2.88)	0.600	1.05 (0.38, 2.88)	0.926
Antiplatelet agents, n(%)				
No	*Ref*		*Ref*	
Yes	1.39 (0.96, 2.00)	0.079	1.16 (0.78, 1.71)	0.461

Abbreviations: SBP, systolic blood pressure; DBP, diastolic blood pressure; BMI, body mass index; Hcy, homocysteine; TC, total cholesterol; TG, total triglyceride; HDL-C, high-density lipoprotein cholesterol; LDL-C, low-density lipoprotein cholesterol; AST, aspartate aminotransferase; ALT, alanine aminotransferase; DM, diabetes mellitus; DM, diabetes mellitus.

Supplement Table 2 Clinical characteristics of the study population grouped by gender

Characteristics	Total subjects	Sex		*P-value*
		Male	Female	
Number of subjects (n)	9839	4548	5291	
Age (years)	63.14 ± 8.99	63.08 ± 9.36	63.19 ± 8.65	0.642
SBP (mmHg)	148.46 ± 17.56	146.61 ± 17.64	150.05 ± 17.33	<0.001
DBP (mmHg)	89.38 ± 10.55	90.85 ± 10.80	88.12 ± 10.18	<0.001
HR (times/min)	76.31 ± 13.99	74.24 ± 13.66	78.09 ± 14.02	<0.001
BMI (kg/m^^{2})	23.65 ± 3.84	23.47 ± 4.06	23.81 ± 3.64	<0.001
BMI group (kg/m^^{2})				<0.001
Control (<24)	5517 (56.07)	2623 (57.67)	2894 (54.70)	
Overweight (≥24, <28)	3304 (33.58)	1511 (33.22)	1793 (33.89)	
General obesity (≥28)	1018 (10.35)	414 (9.10)	604 (11.42)	
WHR	0.91 ± 0.18	0.92 ± 0.25	0.91 ± 0.08	<0.001
Smoking status, n(%)				<0.001
Never	5743 (58.38)	943 (20.73)	4800 (90.74)	
Former	1510 (15.35)	1307 (28.74)	203 (3.84)	
Current	2585 (26.28)	2298 (50.53)	287 (5.43)	
Drinking status, n(%)				<0.001
Never	6135 (62.37)	1626 (35.76)	4509 (85.24%)	
Former	1397 (14.20)	935 (20.56)	462 (8.73%)	
Current	2305 (23.43)	1986 (43.68%)	319 (6.03%)	
Hcy (μmol/L)	14.65 (12.32-18.17)	16.01 (13.34-20.13)	13.59 (11.72-16.48)	<0.001
FBG (mmol/L)	6.16 ± 1.60	6.06 ± 1.49	6.24 ± 1.68	<0.001
TC (mmol/L)	5.16 ± 1.10	4.95 ± 1.04	5.34 ± 1.11	<0.001
TG (mmol/L)	1.45 (1.03-2.13)	1.31 (0.92-1.98)	1.57 (1.15-2.23)	<0.001

HDL-C (mmol/L)	1.60 ± 0.43	1.56 ± 0.45	1.63 ± 0.42	<0.001
LDL-C (mmol/L)	3.00 ± 0.80	2.87 ± 0.77	3.11 ± 0.81	<0.001
SUA (umol/L)	402.79 ± 113.72	449.98 ± 112.19	362.24 ± 98.35	<0.001
HUA, n(%)	4990 (50.72)	2584 (56.82)	2406 (45.47)	<0.001
BUN (mmol/L)	5.25 ± 1.46	5.34 ± 1.49	5.16 ± 1.44	<0.001
Serum creatinine (mmol/L)	64.12 ± 17.23	74.72 ± 15.27	55.02 ± 13.12	<0.001
eGFR (ml/min/1.73m^2)	93.37 ± 14.59	91.43 ± 14.33	95.04 ± 14.61	<0.001
Total bilirubin (mmol/L)	13.40 (10.40-17.60)	14.30 (11.20-18.90)	12.70 (9.80-16.40)	<0.001
Direct bilirubin (mmol/L)	5.20 (4.20-6.60)	5.70 (4.50-7.20)	4.90 (3.90-6.10)	<0.001
AST (U/L)	24.00 (20.00-30.00)	25.00 (21.00-31.00)	24.00 (20.00-29.00)	<0.001
ALT (U/L)	17.00 (13.00-24.00)	18.00 (13.00-26.00)	16.00 (12.00-22.00)	<0.001
DM, n(%)	1723 (17.51)	719 (15.81)	1004 (18.98)	<0.001
Dyslipidemia, n(%)	3608 (36.67)	1458 (32.06)	2150 (40.64)	<0.001
Atrial fibrillation, n(%)	252 (2.56)	109 (2.40)	143 (2.70)	0.338
Antihypertensive agents, n(%)	6338 (64.42)	2906 (63.90)	3432 (64.88)	0.311
Hypoglycemic agents, n(%)	482 (4.90)	194 (4.27)	288 (5.44)	0.007
Lipid-lowering agents, n(%)	324 (3.29)	154 (3.39)	170 (3.21)	0.631
Antiplatelet agents, n(%)	368 (3.74)	191 (4.20)	177 (3.35)	0.026
Ankle brachial index	1.09 ± 0.09	1.10 ± 0.10	1.09 ± 0.08	<0.001
PAD, n(%)	263 (2.67)	144 (3.17)	119 (2.25)	0.005

Abbreviations: SBP, systolic blood pressure; DBP, diastolic blood pressure; HR, heart rate; BMI, body mass index; WHR, waist hip rate; Hcy, homocysteine; FBG, fasting blood glucose; TC, total cholesterol; TG, total triglyceride; HDL-C, high-density lipoprotein cholesterol; LDL-C, low-density lipoprotein cholesterol; SUA, serum uric acid; BUN, blood urea nitrogen; eGFR, estimated glomerular filtration rate; AST, aspartate aminotransferase; ALT, alanine aminotransferase; DM, diabetes mellitus; PAD, peripheral arterial disease.

Supplementary Table 3 Hazard ratios of serum uric acid level categories for peripheral arterial disease in different models

Variables	Event, n(%)	Crude Model		Model I		Model II	
		HR (95%CI)	P-value	HR (95%CI)	P-value	HR (95%CI)	P-value
SUA							
Per SD μmol/L increase	263 (2.67%)	1.00 (1.00, 1.00)	0.004	1.00 (1.00, 1.00)	0.025	1.00 (1.00, 1.00)	0.055
P for interaction of sex		0.187		0.698		0.882	
HUA							
No	109 (2.25%)	Ref		Ref		Ref	
Yes	154 (3.09%)	1.38 (1.08, 1.78)	0.010	1.35 (1.05, 1.74)	0.018	1.33 (1.01, 1.75)	0.045
P for interaction of sex		0.504		0.334		0.674	
Tertiles of SUA							
T1 [38.00, 344.00]	64 (1.96%)	Ref		Ref		Ref	
T2 [345.00, 439.00]	84 (2.55%)	1.31 (0.94, 1.82)	0.111	1.18 (0.84, 1.65)	0.349	1.15 (0.81, 1.63)	0.443
T3 [440.00, 1056.00]	115 (3.50%)	1.81 (1.33, 2.47)	<0.001	1.66 (1.19, 2.31)	0.003	1.69 (1.17, 2.46)	0.005
P for trend		<0.001		<0.001		<0.001	
P for interaction of sex		0.907		0.079		0.262	

Abbreviations: PAD, peripheral arterial disease; SUA, serum uric acid; HUA, hyperuricemia; Ref, reference; HR, hazard ratio; CI, confidence interval; SD, standard deviation.

Model I adjusted for age and sex.

Model II adjusted for age, SBP, DBP, BMI, Hcy, TG, HDL-C, LDL-C, serum creatinine, ALT, AST and antihypertensive agents, lipid-lowering agents and antiplatelet agent.

Supplementary Table 4 Hazard ratios of hyperuricemia for peripheral arterial disease in different models

Variables	Event, n(%)	Crude Model		Model I		Model II	
		HR (95%CI)	*P-value*	*HR (95%CI)*	*P-value*	*HR (95%CI)*	*P-value*
HUA							
No	43 (1.9%)	*Ref*		*Ref*		*Ref*	
Yes	220 (2.9%)	1.51 (1.08, 2.10)	0.015	1.36 (0.97, 1.90)	0.076	1.30 (0.91, 1.87)	0.147
P for trend		0.015		0.076		0.143	
P for interaction of sex		0.786		0.514		0.399	

Abbreviations: PAD, peripheral arterial disease; SUA, serum uric acid; HUA, hyperuricemia; *Ref*, reference; *HR*, hazard ratio; *CI*, confidence interval; SD, standard deviation.

Model Ⅰ adjusted for age and sex.

Model Ⅱ adjusted for age, sex,SBP, DBP, BMI, Hcy, HDL-C, LDL-C, TC,TG,serum creatinine, ALT, AST and antihypertensive agents, lipid-lowering agents, antiplatelet agent,total bilirubin,direct bilirubin,estimated glomerular filtration rate,WHR and BUN.

Supplementary Table 5 Hazard ratios of hyperuricemia for peripheral arterial disease by sex in different models

Variables	Event, n(%)	Crude Model		Model I		Model II	
		HR (95%CI)	*P-value*	*HR (95%CI)*	*P-value*	*HR (95%CI)*	*P-value*
Male							
HUA							
No	14 (2.2%)	*Ref*		*Ref*		*Ref*	
Yes	130 (3.3%)	1.49 (0.85, 2.60)	0.161	1.57 (0.90, 2.76)	0.114	1.78 (0.97, 3.25)	0.062
P for trend		0.161		0.114		0.063	
Female							
HUA							
No	29 (1.8%)	*Ref*		*Ref*		*Ref*	
Yes	90 (2.4%)	1.35 (0.89, 2.06)	0.161	1.26 (0.83, 1.93)	0.282	1.00 (0.63, 1.60)	0.991
P for trend		0.161		0.282		0.921	

Abbreviations: PAD, peripheral arterial disease; SUA, serum uric acid; HUA, hyperuricemia; *Ref*, reference; *HR*, hazard ratio; *CI*, confidence interval; SD, standard deviation.

Model Ⅰ adjusted for age.

Model Ⅱ : adjusted for age, sex,SBP, DBP, BMI, Hcy, HDL-C, LDL-C, TC,TG,serum creatinine, ALT, AST and antihypertensive agents, lipid-lowering agents, antiplatelet agent,total bilirubin,direct bilirubin,estimated glomerular filtration rate,WHR and BUN.

Supplementary Table 6 Hazard ratios of hyperuricemia for peripheral arterial disease by sex in different models

Variables	Event, n(%)	Crude Model		Model I		Model II	
		HR (95%CI)	*P-value*	*HR (95%CI)*	*P-value*	*HR (95%CI)*	*P-value*
Male							
HUA							
No	51 (2.6%)	*Ref*		*Ref*		*Ref*	
Yes	93 (3.6%)	1.40 (0.99, 1.98)	0.057	1.54 (1.09, 2.19)	0.016	1.85 (1.18, 2.92)	0.008
P for trend		0.057		0.016		0.008	
Female							
HUA							
No	58 (2.0%)	*Ref*		*Ref*		*Ref*	
Yes	61 (2.5%)	1.34 (1.04, 1.71)	0.202	1.20 (0.83, 1.73)	0.328	1.03 (0.65, 1.63)	0.890
P for trend		0.202		0.328		0.890	

Abbreviations: PAD, peripheral arterial disease; SUA, serum uric acid; HUA, hyperuricemia; *Ref*, reference; *HR*, hazard ratio; *CI*, confidence interval; SD, standard deviation.

Model Ⅰ adjusted for age.

Model Ⅱ : adjusted for age, sex,SBP, DBP, BMI, Hcy, HDL,LDL,TG, meat, fruit, estimated glomerular filtration rate and urea.

Supplementary Figure 1: The Effect Size of HUA on the Prevalence of PAD in Males

Subgroup	Events, n(%)	OR (95% CI)	P-value	Interactive P
**Age (years), dichotomy**				**0.985**
<60	16 (1.11%)	1.71 (0.51, 5.74)	0.385	
≥60	128 (4.11%)	1.68 (1.12, 2.53)	0.013	
**SBP (mmHg), dichotomy**				**0.527**
Low [87.00, 146.67]	67 (2.73%)	1.49 (0.85, 2.64)	0.167	
High [147.00, 255.00]	77 (3.68%)	1.92 (1.13, 3.27)	0.016	
**DBP (mmHg), dichotomy**				**0.551**
Low [46.00, 89.33]	95 (4.78%)	1.59 (0.98, 2.56)	0.059	
High [89.67, 130.67]	49 (1.91%)	2.04 (1.03, 4.07)	0.042	
**BMI group (kg/m^2)**				**0.241**
Control (<24)	105 (4.00%)	1.64 (1.05, 2.56)	0.031	
Overweight (≥24, <28)	27 (1.79%)	2.76 (1.06, 7.20)	0.038	
Obesity (≥28)	12 (2.90%)	0.53 (0.10, 2.74)	0.450	
**Smoking status**				**0.371**
Never	21 (2.23%)	3.55 (1.05, 11.94)	0.041	
Former smoker	32 (2.45%)	1.66 (0.71, 3.90)	0.241	
Current smoker	91 (3.96%)	1.43 (0.88, 2.33)	0.149	
**Drinking status**				**0.831**
Never	64 (3.94%)	2.01 (1.10, 3.67)	0.023	
Former	33 (3.53%)	1.53 (0.66, 3.56)	0.319	
Current	47 (2.37%)	1.60 (0.81, 3.13)	0.175	
**Antihypertensive agents**				**0.389**
No	42 (2.56%)	2.32 (1.09, 4.93)	0.029	
Yes	102 (3.51%)	1.58 (1.00, 2.50)	0.051	
**Homocysteine (μmol/L)**				**0.535**
<15	42 (2.25%)	1.46 (0.72, 2.95)	0.294	
≥15	102 (3.80%)	1.90 (1.19, 3.04)	0.007	
**LDL-C (mmol/L), dichotomy**				**0.863**
Low [0.33, 2.93]	76 (2.95%)	1.79 (1.06, 3.02)	0.030	
High [2.94,8.44]	68 (3.44%)	1.67 (0.94, 2.97)	0.082	

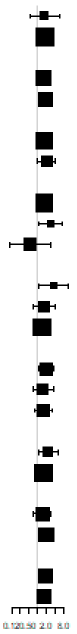

Abbreviations: Each stratification adjusted for age, SBP, DBP, BMI, smoking and drinking status, Hcy, TG, LDL-C, serum creatinine, ALT, AST, antihypertensive agents and antiplatelet agents except the subgroup variable. Abbreviations: Ref = reference; HR = hazard ratio; CI = confidence interval; SBP, systolic blood pressure; DBP, diastolic blood pressure; BMI, body mass index; LDL-C, low-density lipoprotein cholestero

Supplementary Figure 2: The Effect Size of HUA on the Prevalence of PAD in Females

Subgroup	Events, n(%)	OR (95% CI)	P-value	Interactive P
**Age (years), dichotomy**				**0.196**
<60	26 (1.54%)	0.63 (0.26, 1.54)	0.310	
≥60	93 (2.58%)	1.23 (0.77, 1.95)	0.384	
**SBP (mmHg), dichotomy**				**0.612**
Low [83.33, 146.67]	42 (1.75%)	0.89 (0.45, 1.76)	0.729	
High [147.00, 226.67]	77 (2.66%)	1.10 (0.67, 1.82)	0.698	
**DBP (mmHg), dichotomy**				**0.746**
Low [41.67, 89.33]	81 (2.79%)	1.09 (0.66, 1.79)	0.737	
High [89.67, 142.33]	38 (1.59%)	0.96 (0.47, 1.98)	0.910	
**BMI group (kg/m^2)**				**0.778**
Control (<24)	70 (2.42%)	1.05 (0.62, 1.80)	0.853	
Overweight (≥24, <28)	33 (1.84%)	0.81 (0.38, 1.74)	0.592	
Obesity (≥28)	16 (2.65%)	1.30 (0.38, 4.45)	0.675	
**Antihypertensive agents**				**0.465**
No	38 (2.05%)	0.81 (0.39, 1.68)	0.574	
Yes	81 (2.36%)	1.13 (0.68, 1.85)	0.640	
**Antiplatelet agents**				**0.615**
No	111 (2.17%)	1.06 (0.70, 1.62)	0.769	
Yes	8 (4.52%)	0.60 (0.07, 5.47)	0.651	
**Homocysteine (μmol/L)**				**0.911**
<15	68 (2.00%)	1.02 (0.59, 1.74)	0.951	
≥15	51 (2.69%)	1.07 (0.56, 2.02)	0.844	
**LDL-C (mmol/L), dichotomy**				**0.261**
Low [0.61, 2.94]	49 (2.11%)	0.56 (0.29, 1.07)	0.077	
High [2.94,8.03]	70 (2.36%)	1.58 (0.93, 2.68)	0.089	
**AST (U/L), dichotomy**				**0.829**
Low [2.16, 23.00]	70 (2.68%)	1.09 (0.64, 1.84)	0.762	
High [24.00,220.00]	49 (1.83%)	0.99 (0.52, 1.86)	0.963	

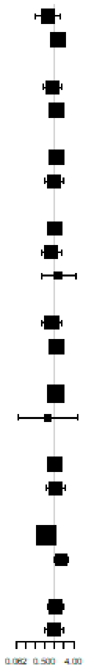

Abbreviations: Each stratification adjusted for age, SBP, DBP, BMI, Hcy, TG, HDL-C, LDL-C, serum creatinine, ALT, AST, antihypertensive agents, lipid-lowering agents and antiplatelet agents except the subgroup variable. Abbreviations: Ref = reference; HR = hazard ratio; CI = confidence interval; SBP, systolic blood pressure; DBP, diastolic blood pressure; BMI, body mass index; LDL-C, low-density lipoprotein cholesterol; AST, aspartate aminotransferase.
